# Maternal Gestational Cortisol and Testosterone Are Associated with Trade-Offs in Offspring Sex and Number in a Free-Living Rodent (*Urocitellus richardsonii*)

**DOI:** 10.1371/journal.pone.0111052

**Published:** 2014-10-29

**Authors:** Calen P. Ryan, W. Gary Anderson, Charlene N. Berkvens, James F. Hare

**Affiliations:** 1 Department of Biological Sciences, University of Manitoba, Winnipeg, Manitoba, Canada; 2 Assiniboine Park Zoo, Winnipeg, Manitoba, Canada; University of Turku, Finland

## Abstract

The adaptive manipulation of offspring sex and number has been of considerable interest to ecologists and evolutionary biologists. The physiological mechanisms that translate maternal condition and environmental cues into adaptive responses in offspring sex and number, however, remain obscure. In mammals, research into the mechanisms responsible for adaptive sex allocation has focused on two major endocrine axes: the hypothalamic pituitary adrenal (HPA) axis and glucocorticoids, and the hypothalamic pituitary gonadal (HPG) axis and sex steroids, particularly testosterone. While stress-induced activation of the HPA axis provides an intuitive model for sex ratio and litter size adjustment, plasma glucocorticoids exist in both bound and free fractions, and may be acting indirectly, for example by affecting plasma glucose levels. Furthermore, in female mammals, activation of the HPA axis stimulates the secretion of adrenal testosterone in addition to glucocorticoids (GCs). To begin to untangle these physiological mechanisms influencing offspring sex and number, we simultaneously examined fecal glucocorticoid metabolites, free and bound plasma cortisol, free testosterone, and plasma glucose concentration during both gestation and lactation in a free-living rodent (*Urocitellus richardsonii*). We also collected data on offspring sex and litter size from focal females and from a larger study population. Consistent with previous work in this population, we found evidence for a trade-off between offspring sex and number, as well as positive and negative correlations between glucocorticoids and sex ratio and litter size, respectively, during gestation (but not lactation). We also observed a negative relationship between testosterone and litter size during gestation (but not lactation), but no effect of glucose on either sex ratio or litter size. Our findings highlight the importance of binding proteins, cross-talk between endocrine systems, and temporal windows in the regulation of trade-offs in offspring sex and number.

## Introduction

Since its inception, adaptive sex allocation (ASA) has been of great interest to evolutionary biologists and ecologists, but has largely focused on the environmental conditions favoring the production of offspring of one sex over the other [1]–[4]. While Trivers and Willard’s hypothesis has seen theoretical and empirical support [5]–[7], workers have also expanded the original idea to accommodate the diverse ecology and life histories of the organisms in which ASA has been studied [3]. Progress disentangling the growing number of hypotheses for ASA, however, has been slowed by our rudimentary understanding of the physiological mechanisms through which adaptive manipulation of sex ratio putatively occurs [8]. In mammals, several physiological processes linking maternal cues to offspring sex ratio have been proposed, each involving the translation of ecological, social, and physiological information into neuroendocrine signals that ultimately affect fertilization, implantation, growth, or survival of developing embryos [9]–[14]. While there may be important phylogenetic differences in mechanisms for ASA [15], work in this area has centered around two major endocrine axes.

The first, involving glucocorticoids (GCs, cortisol and/or corticosterone) and the hypothalamic-pituitary adrenal (HPA) axis, was founded on observations that social and environmental stressors were linked to sex ratio in both birds [16], [17] and mammals [14], [18]. Given their role in reproduction, embryonic development, and immune function, GCs are promising candidates for the translation of ecological and physiological cues into ASA [19]–[21]. One potential mechanism for ASA in mammals involves increased mortality of female embryos resulting from GC-induced elevations in circulating glucose [11]. Yet while the ‘glucose-metabolism’ hypothesis has seen empirical support ([22], [23], but also see [24]), it predicts that mothers with high GCs should have more males, and therefore could be at odds with the predictions of the Trivers-Willard hypothesis in its original form [25]. Additionally, concurrent associations between GCs, glucose, sex ratio – and in the case of polytocous species – litter size, have not been reported. To further complicate matters, glucocorticoids exist in distinct physiological fractions (free hormone and hormone bound to the carrier protein, cortisol binding globulin, or CBG). There is ongoing interest and debate (e.g. [26]) about the function of free versus bound glucocorticoids, their biological relevance to downstream physiological metrics [27], and their effects on individual life history strategies [28]. This additional layer of complexity could prove necessary for understanding potential mechanisms connecting GCs and sex ratio.

The second endocrine axis postulated to affect sex ratio involves the hypothalamic-pituitary gonadal (HPG) axis and sex steroids, particularly testosterone (T). Maternal social dominance, intrauterine environment, and menstrual phase at insemination, all affecting circulating maternal T, have been associated with biases in sex ratio [13], [29], [30]. Moreover, testosterone measured in serum [23] and ovular follicular fluid [31] have themselves been directly associated with sex ratio. Yet considerable empirical work has failed to support any relationship between measures of testosterone and ASA [32]–[35] – at the very least raising questions about the universality of testosterone-mediated manipulation of offspring sex ratio. The equivocal support for T-mediated ASA is also confounded by the fact that T secretion in female mammals, the source of which is predominantly the adrenal glands, can be stimulated by adrenocorticotropic hormone (ACTH) via the activation of the HPA axis [12], [36].

The present study examines ASA in Richardson’s ground squirrels (*Urocitellus richardsonii*), a semi-fossorial, colonial rodent of the northern North American plains [37]. Previous work on this species revealed a positive relationship between litter sex ratio and maternal fecal glucocorticoid metabolites (FGMs) during gestation (a measure of ‘stress’ or ‘allostatic load’) as well as a negative relationship between offspring number and sex ratio ([25], but see [38]). These findings collectively support the glucose metabolism hypothesis; however *plasma* cortisol, glucose, and testosterone are necessary to: (a) distinguish between the competing physiological mechanisms for ASA described above; and (b) to refine our understanding of the timing of the operative window of such a mechanism. To address these questions in the current study, we measured litter size and sex ratio, as well as plasma cortisol (bound and free), glucose, testosterone (free), and FGMs simultaneously at four distinct time points: early gestation, late gestation, early lactation, and late lactation. For robustness, we also collected data on litter size, juvenile emergence, and sex ratio from additional individuals in our larger study population. Support for the glucose metabolism hypothesis would involve a positive relationship between plasma cortisol and glucose, and a positive and negative relationship between these measures and sex ratio and litter size, respectively. Alternatively, if activation of the HPA affects sex ratio indirectly through the stimulation of adrenal testosterone, we predicted positive relationships between plasma cortisol, testosterone, and sex ratio.

## Methods and Materials

### Study site and sample collection

This study was conducted on free-living female Richardson’s ground squirrels (*Urocitellus richardsonii*) residing within the grounds of the Assiniboine Park Zoo in Winnipeg, Manitoba (49°52′N, 97°14′W). We began observations on 10 April 2013 as female squirrels emerged from hibernation and began mating. Breeding date was based on direct observation of a copulatory plug or semen within the vagina for over half (51/94) of the females within our study area, and could be inferred by back calculation based on juvenile emergence for another 13 females. The focal females we sampled for physiological measures comprised a subset of 21 females within the study area with unambiguous breeding dates. Using breeding date and the duration of gestation (23 days, [39]) and lactation (29 days, [40]) for this species, sampling was divided into four roughly evenly spaced intervals (early and late gestation and early and late lactation–6, 19, 33 and 46 days following breeding). For sampling, females were trapped by placing Tomahawk live traps (Tomahawk Live Trap Co., Tomahawk, WI) baited with peanut butter (No Name Smooth Peanut Butter; Loblaws Inc., Toronto, Ontario, Canada) at or near burrow entrances. Females were then immediately transferred to an adjacent facility where 0.3–0.4 ml of blood was collected from the lateral saphenous vein of each subject using a sterile 0.3 ml syringe and 29 gauge ½ inch needle (Insulin Syringe U-100, Becton Dickinson Co., Franklin Lakes New Jersey). The sampling protocol was the same for all individuals, but required more time than the 3–5 minute window typical for ‘baseline’ glucocorticoid measurements [19]. A separate analysis found that total plasma cortisol and free cortisol were significantly higher for the sampling protocol described here than from baseline samples taken within 3 minutes, however bound cortisol, testosterone, and glucose were not [41]. Blood was centrifuged at 13,000 g for 5 minutes, and plasma was decanted with a micropipette. Any uncontaminated fecal samples produced at this time were also collected using disposable wooden sticks, and fecal and plasma samples were frozen in liquid nitrogen until transfer to −80°C for storage. Prior to release at their point of capture, we applied commercially available hair dye (Clairol Hydrience 52S; Pearl Black, Stamford, CT) to the dorsal pelage of the animals in unique patterns to facilitate individual identification. All females had been previously and permanently identified by inserting numbered metal ear tags (National Band and Tag Company, Monel no. 1, Newport, KY) through the pinna of the right ear.

*U. richardsonii* females utilize and defend small core areas with little overlap with the core areas of other females during pregnancy and lactation [42]. The offspring belonging to a given mother were therefore determined by which burrow the litter emerged from and when. While we cannot rule out sex differential secondary mortality, the number of embryos in this species is closely associated with the number of emerging young, infanticide is rare, and our litter sizes are comparable to those observed at birth in other studies [37]. Using previously described methods [25], young of the year were trapped, sexed, marked and released, typically within 2 days of their initial emergence from their natal burrow. When all pups in a litter were accounted for, we recorded litter sex ratios for each mother in the conventional manner, as a ratio of males to total offspring produced [43]. We were able to determine with accuracy the litter size and sex ratios for 44 breeding females in the study area. Of our focal females, we were unable to accurately determine litter size for one female, and 6 females failed to successfully rear young to emergence from the maternal burrow, restricting the sample size for certain comparisons.

### Ethics Statement

This study was approved by the University of Manitob’s Fort Garry Campus Protocol Management and Review Committee (Protocol Number: F12-014) and by the Animal Ethics Review Panel of the Assiniboine Park Zoo, which adhere to the recommendations for the ethical treatment of animals in research set forth by the Canadian Council on Animal Care, the Animal Behavior Society, and the American Society of Mammalogists.

### Laboratory Procedures

Extraction and measurement of fecal samples followed previously published protocols [25], [41], with minor alterations. Samples were dried overnight at 60°C then 0.5 ml of 95% ethanol was added to approximately 0.1 g of dried and ground fecal matter. Samples were then mixed vigorously, centrifuged at 4°C for 10 min at 13, 000 g. Supernatant was removed and a known volume was then transferred to an assay tube. This process was repeated, the extract pooled, and the ethanol was evaporated using a sample concentrator (Savant, Thermo-Fisher). The resulting pellet was resuspended in RIA buffer (0.1 M phosphate buffer, 0.9% NaCl (w/v) and 0.5% bovine serum albumin (w/v)) immediately prior to measurement in a RIA. Measurement of cortisol and testosterone from plasma samples followed similar procedures; however plasma cortisol extraction involved an initial dilution of 10 µl of raw plasma into 1 ml of ethanol prior to two separate washes of 80 µl of diluted sample with 500 µl of 95% ethanol. Similar procedures were used for plasma testosterone extraction, but without an initial dilution step. As for the fecal extractions, samples were then mixed vigorously before centrifugation at 4°C for 10 minutes at 13, 000 g. The supernatants from both washes were combined and dried down in a sample concentrator and the resulting pellet was re-suspended and diluted as necessary in RIA buffer just prior to measurement as described above.

For the cortisol and testosterone assays, 100 µl of re-suspended sample was combined with 100 µl of antigen-specific antibody (1∶16,000 and 1:75,000 dilution for cortisol and testosterone, respectively, both Fitzgerald Industries, NY, USA) and 100 µl (5,000 dpm) of tritiated antigen (GE Healthcare, NJ, USA). Assay tubes were allowed to incubate at room temperature for 1 h, and then overnight at 4°C. Addition of 100 µl of dextran-coated charcoal to all assay tubes (0.5% w/v dextran and 5% w/v charcoal) followed by incubation at 4°C for 15 min terminated the assay. Tubes were then centrifuged at 4°C for 30 min at 2500 g to separate bound from unbound ligand. The resulting supernatant was decanted into 7 ml scintillation vials and 4 ml of liquid scintillation cocktail (Ultima Gold AB, Perkin Elmer, MA USA) was added to each tube. Radioactivity was measured using a liquid scintillation counter (Tri-Carb 3110TR, Perkin Elmer, MA, USA) and cortisol and testosterone concentrations were interpolated from standard curves using known concentrations of cold ligand. All samples were processed in duplicate and standards were processed in triplicate. Inter- and intra-assay coefficients of variation for the testosterone assay were 4.0% and 7.4%, respectively, with a lower detection limit of 0.10 ng/ml. Extraction efficiency for T was 101.4±7.5%, and all values falling within the fitted range of each curve were used. Assay parameters for FGMs and plasma cortisol are described elsewhere [41], [44]. Serial dilutions for FGMs, plasma cortisol, and testosterone ran parallel to the standard curve.

To measure corticosteroid-binding globulin (CBG), endogenous cortisol was stripped from each sample by incubation with 2 parts volume of dextran-coated charcoal solution (0.1% Dextran, 1% activated charcoal in 50 mM Tris assay buffer, pH 7.4) for 30 minutes at room temperature, followed by centrifugation (2,500 g) for 10 min at 4°C. Fifty microliters of stripped plasma was combined with 50 µl 16.7 nM [^3^H]-Cortisol and 50 µl 50 mM Tris buffer (total count) or 3 µM cold cortisol (non-specific binding), covered and incubated overnight at 4°C (for a final dilution of 1∶405). Bound [^3^H]-Cortisol was separated from unbound labeled hormone by vacuum filtration through glass fiber filters (Whatman GF/B, pre-soaked in 0.3% PEI in 25 mM Tris buffer) using a Brandel cell harvester (Model: M24, Gaithersburg, MD), with three 3 ml rinses of ice-cold 25 mM Tris buffer. The disassociation constant (K_D_) for this species was 3.18±1.33 nM, determined from the average of two separate assays using pooled plasma and [^3^H]-Cortisol concentrations between 0.31–30 nM or 3 µM cold cortisol in 25 mM Tris buffer.

Glucose was measured, in duplicate, using a commercially available *in* *vitro* assay kit (Wako Diagnostics, Richmond, VA), with the intra and inter-assay coefficients of variation of 17.5% and 7.6%, respectively.

### Statistical Analyses

Prior to statistical analysis, data (publicly available in figshare database: http://dx.doi.org/10.6084/m9.figshare.1067068), were assessed for outlying data points, normality, and missing values. Highly skewed data for fecal cortisol, free cortisol, plasma glucose, and plasma testosterone were ultimately log-transformed to meet model assumptions. Analyses involving sex ratio employed generalized linear models with a binomial family, weighted by litter size. For other analyses, mixed effects models with female identity as a random factor were used to account for multiple measurements per individual. When multiple data points predicted a single outcome from one female (e.g. sex ratio), calculation of the covariance matrix required switching the dependant and independent variables. As an independent variable, sex ratio was arcsine square root transformed to improve residual structure and truncate predicted values near 0 and 1. Mixed effects models were assessed for temporal autocorrelation, run with and without random slopes and with and without weighted variances for sample, where applicable. Based on the lowest AIC values, adding a correlated error structure or random slopes did not improve model fit, but in certain instances weighted variances did (i.e. variance differed between early and late gestation and lactation), and so were included when this was the case.

To make meaningful comparisons between this study and our previous work in the same population [25], we reanalyzed those data using GLMs, as described above. In every case, the relationships we described in that study were robust when using these methods. In this study, interactions, such as sample time and breeding period, were retained when significant and otherwise discarded from the final result. All analyses were followed with standard model validation diagnostics (e.g. normality, heteroscedasticity of residuals). Residuals with high leverage and Cook’s distance (>4/n), or with standardized values outside the 95% confidence interval for fitted values were examined carefully as potentially influential outliers. Model fit was often improved by data transformation, weighted variances, or as a last resort, by removing influential data points and rerunning the analysis. Models that showed signs of violating test assumptions or that required point deletion were rerun using non-parametric tests (e.g. Spearman’s rank-order correlation), with results reported if the outcome of both tests differed. Missing values (e.g. due to insufficient plasma for all assays) or data outside assay detection limits resulted in differences in the number of observations between models, but in all cases statistical tests used the maximum available number of data points. Statistical tests were generally derived from *a priori* predictions described in the Introduction, except when controlling for potentially confounding factors (e.g. age) or where particularly informative, such as in the absence of relationships seen in gestation during lactation. As a result, and due to the prohibitively high risk of type II error [45], [46], corrections for multiple comparisons were not applied.

## Results

### Physiological parameters throughout the breeding season

Total plasma cortisol decreased throughout the breeding season, with significantly lower levels during lactation compared to early gestation (Table 1). Similarly, both maximum bound cortisol and FGMs decreased during breeding, with levels dropping significantly by late lactation (Table 1). Free cortisol levels, however, did not change significantly throughout the season (Table 1). Plasma testosterone was highest during early lactation, dropping to the lowest levels by late lactation, though these differences were non-significant (Table 1). Glucose levels were highest during early gestation, and dropped significantly by late lactation (Table 1).

**Table 1:** Physiological parameters for 21 female Richardson’s ground squirrels through the breeding season.

PhysiologicalParameter	Breeding Period				Test_df_	*P*-value
	EarlyGestation	LateGestation	EarlyLactation	LateLactation		
Total PlasmaCortisol(ng⋅mL^−1^)Θ	182.43±15.09^a^	182.32±14.83^ab^	130.64±10.31^c^	137.45±7.67^bc^	n = 76;χ^2^_3_ = 15.39	*P* = 0.002
BoundPlasmaCortisol(ng⋅mL^−1^)Θ	121.37±15.94^ab^	144.02±14.83^a^	96.71±10.31^bc^	86.35±7.59^bc^	n = 74;χ^2^_3_ = 17.08	*P*<0.001
Free PlasmaCortisol(ng⋅mL^−1^)Θ	8.94(3.57–132.01)	9.05(1.48–57.32)	14.95(2.27–50.59)	50.07(8.87–91.68)	n = 74;χ^2^_3_ = 1.90	*P* = 0.593
FecalGlucocorticoidMetabolites(FGMs)‡(ng⋅g^−1^)	14.98^a^(4.61–20.71)	13.74^a^(8.29–23.09)	7.36^a^(3.44–15.42)	3.32^a^(2.22–11.17)	n = 76;χ^2^_3_ = 8.01	*P* = 0.046∞
PlasmaTestosterone‡(pg⋅mL^−1^)	102.42(80.38–187.19)	124.04(87.56–164.6)	174.31(99.19–262.17)	86.09(64.43–96.38)	n = 51;χ^2^_3_ = 6.77	*P* = 0.080
PlasmaGlucose‡(mg⋅dL^−1^)	130.8^a^(121.2–135.9)	114.9^ab^(110.9–127.1)	117.5^ab^(109.8–139.8)	114.1^b^(104.1.61–117.59)	n = 75;χ^2^_3_ = 12.56	*P* = 0.006

Θ Mean ± standard error: total plasma cortisol, bound cortisol.

‡ Median and interquartile range: Free cortisol, FGMs, plasma testosterone, and plasma glucose.

∞ FGMs not significantly different using Tukey post-hoc contrasts.

Means (± SEM) or median and interquartile range, plus overall results of linear mixed effects model and *P*-values, number of observations for each model are shown. For tests with significant overall differences, superscript letters indicate significant differences using post-hoc Tukey contrasts (*P*<0.05).

The proportion of CBG saturated and the proportion of cortisol bound were positively (β = 0.20±0.04 ng/mL, *df* = 49, *t* = 5.09, *P*<0.001; Intercept: 47.01±8.34, *df* = 49, *t* = 5.63, *P*<0.001) and negatively (β = 0.22±0.04 ng/mL, *df* = 49, *t* = −5.21, *P*<0.001; Intercept: 110.63± 10.23, *df* = 49, *t* = 10.23, *P*<0.001) correlated with total plasma cortisol levels, respectively. In other words, CBG was most saturated when total plasma cortisol levels were highest, at which point the proportion of hormone bound was lowest. Total plasma cortisol itself was positively correlated with testosterone (β = 0.21±0.00 pg/mL, *df* = 31, *t* = 2.45, *P* = 0.020; Intercept: 70.5± 13.6 pg/mL, *df* = 31, *t* = −12.42, *P*<0.001). This was also true of the free (β = 0.27±0.12 pg/mL, *df* = 30, *t* = 2.30, *P* = 0.029; Intercept: 86.0± 19.8 pg/mL, *df* = 30, *t* = 24.49, *P*<0.001) but not bound (χ^2^_1_ = 0.98, *P* = 0.322) fractions of cortisol at the time of sampling. There was a negative, but marginally non-significant, correlation between total plasma cortisol and plasma glucose (χ^2^_1_ = 3.72, *P* = 0.054). FGMs were not correlated with total (χ^2^_1_ = 3.33, *P* = 0.068), free (χ^2^_1_ = 0.572, *P* = 0.450), nor bound cortisol (χ^2^_1_ = 0.085, *P* = 0.771), or with testosterone (χ^2^_1_<0.001, *P* = 0.988) or plasma glucose (χ^2^_1_ = 1.18, *P* = 0.276).

### Reproductive traits for the 2013 breeding season

Of the focal females observed breeding, roughly two thirds (67%) successfully raised young that emerged from the maternal burrow (Table 2). This rate was very similar to that of the larger study population (68%). Within the larger population, there was no effect of observed breeding date on litter size (χ^2^_1_ = 0.02, *P* = 0.898) or sex ratio (χ^2^_1_ = 0.002, *P* = 0.968). There was also no significant effect of age on litter size (χ^2^_1_ = 3.03, *P* = 0.387) or litter sex ratio (χ^2^_1_ = 2.91, *P* = 0.406). Litter size was significantly and negatively correlated with litter sex ratio, both within the larger study population (χ^2^_1_ = 9.87, *P* = 0.002) and within the subset of focal females (χ^2^_1_ = 4.10, *P* = 0.043), however post-test diagnostics suggested violations of the model assumptions (e.g. non-normal distribution of residuals, high leverage points) that were not easily remedied. We rerun these analyses using a ranked non-parametric test less affected by these deviations, and found that sex ratio and litter size were uncorrelated in the larger study population (Spearman’s ρ = −0.14, *P* = 0.351), but remained negatively correlated for our group of focal females (Spearman’s ρ = −0.55, *P* = 0.042).

**Table 2:** Reproductive parameters for female Richardson’s ground squirrels during the 2013 breeding season.

Reproductive Parameter	Focal Females		Study Area Population	
	Mean (± SE)	Range	Mean (± SE)	Range
Female age	1.57±0.24	1–5	1.60±0.13	1–5
Breeding date	19 April	18 April–23 April	16 April	10 April–23 April
Juvenile Emergence	10 June	5 June–14 June	8 June	1 June–17 June
Litter Size (litters >0)	5.07±0.65	1–10	5.18±0.26	1–10
No. males	2.29±0.30	1–5	2.59±0.20	0–6
No. females	2.79±0.58	0–7	2.59±0.24	0–7
Sex Ratio‡	0.47 (0.35–0.66)	0.2–1	0.50 (0.32–0.67)	0–1

‡ Sex ratio shown as median and interquartile range.

Physiological measurements were accompanied by reproductive data for focal females only (n = 15), however reliable reproductive data was available for between 44 and 65 females in the larger study area, depending on the variable.

### Physiological metrics and reproductive traits during the breeding season

**Figure 1:**
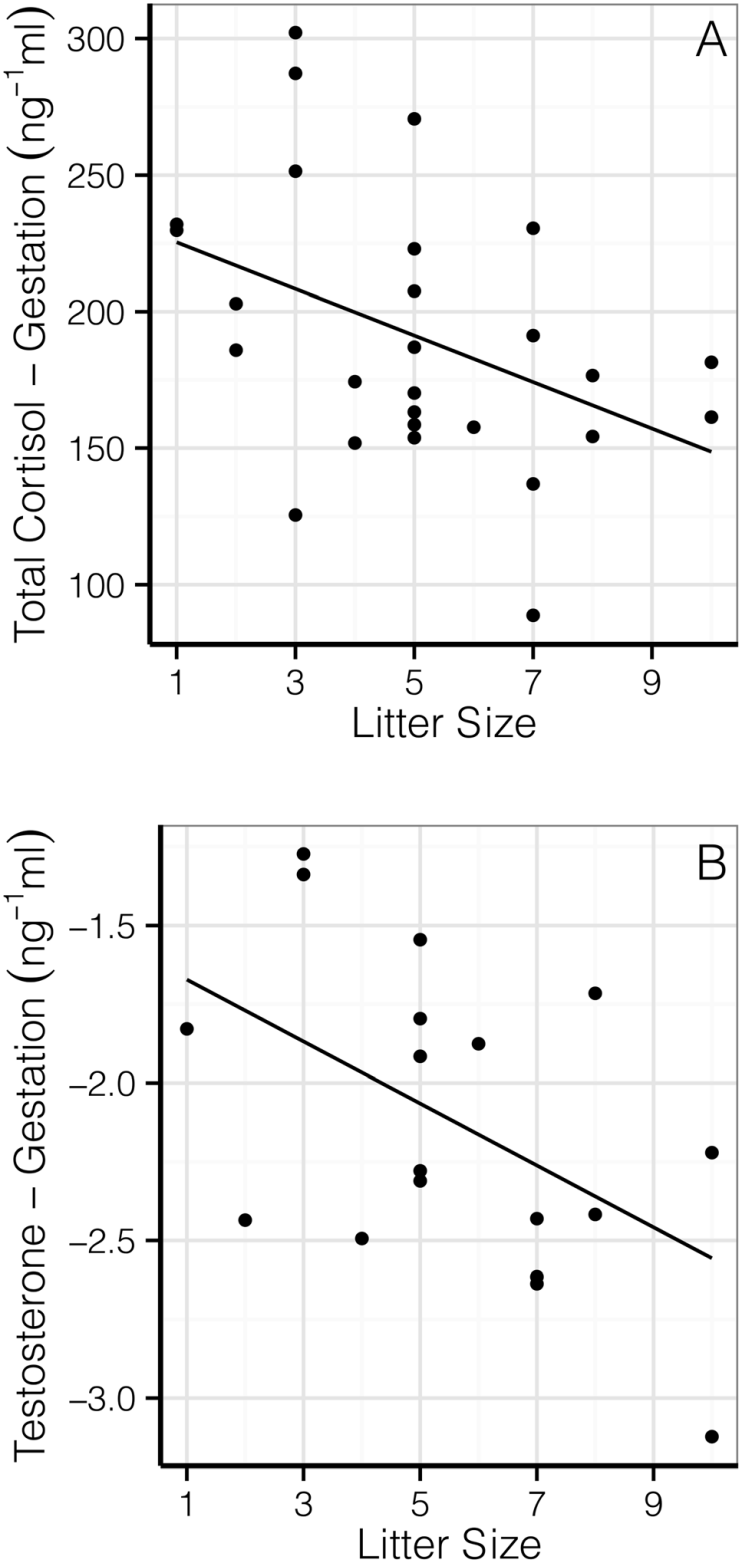
Relationship between total maternal gestational cortisol (A) and testosterone (B) and litter size. Both graphs show multiple points for each individual, accounted for using mixed effects models with maternal identity as a random factor (*P*<0.05 for both).

Litter size was negatively correlated with total plasma cortisol (χ^2^_1_ = 3.81, *P* = 0.051), an effect that was driven by a relationship during gestation (β = 8.54±3.85 ng/mL, *df* = 12, *t* = −2.22, *P* = 0.047; Intercept: 235.94± 21.6 ng/mL, *df* = 12, *t* = −10.91, *P*<0.001; Fig. 1A), but not lactation (χ^2^_1_ = 0.60, *P* = 0.437). Testosterone was also negatively associated with litter size during gestation (β = 17.91±7.91 pg/mL, *df* = 12, *t* = −2.25, *P* = 0.044; Intercept: 205.96± 47.44 pg/mL, *df* = 12, *t* = −6.03, *P*<0.001; Fig. 1B) but not lactation (χ^2^_1_ = 2.44, *P* = 0.119). There were no significant relationships between bound cortisol (χ^2^_1_ = 3.03, *P* = 0.082), FGMs (χ^2^_1_ = 1.22, *P* = 0.269), or glucose (χ^2^_1_ = 0.11, *P* = 0.741) and litter size.

**Figure 2:**
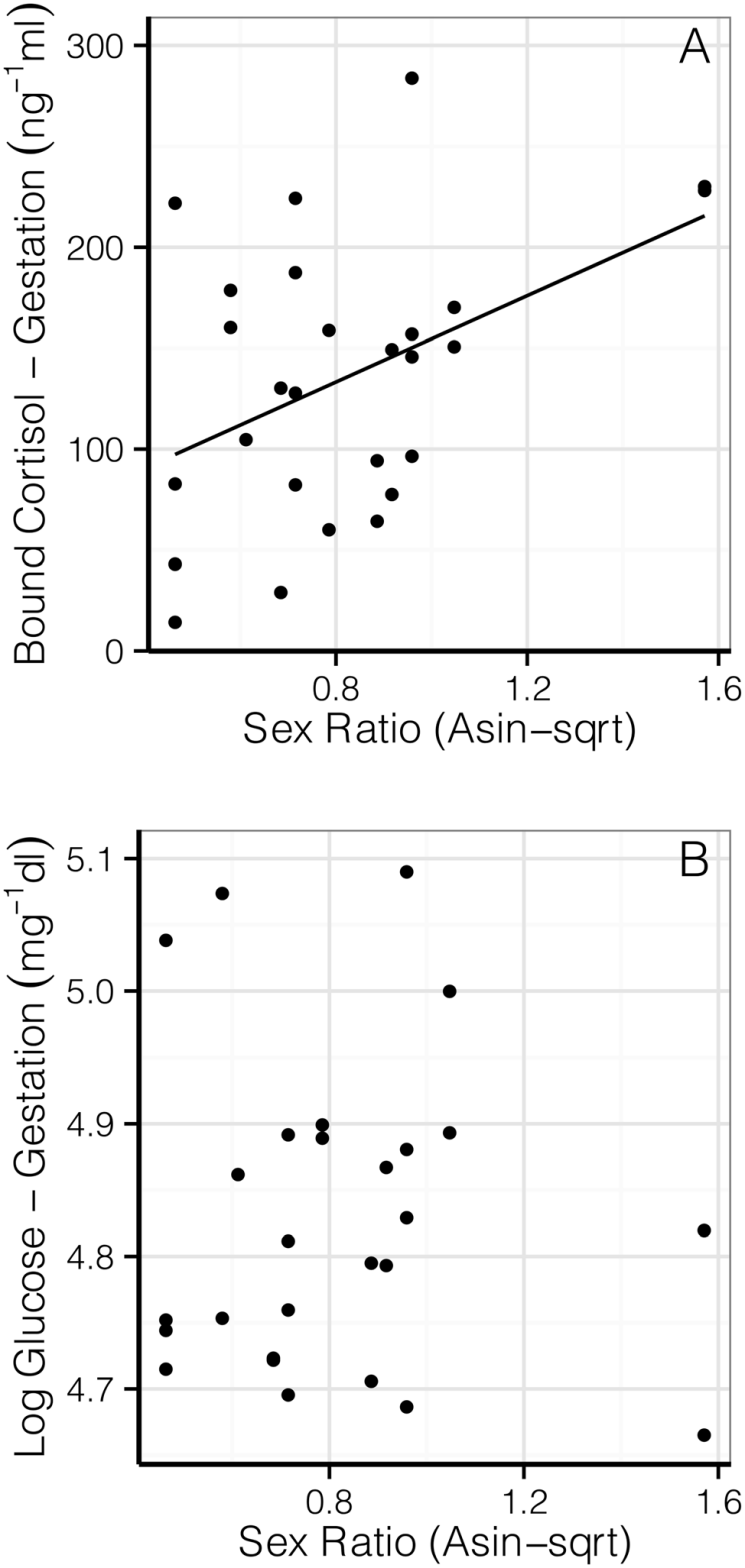
Relationship between bound maternal gestational cortisol (A) and glucose (B) during gestation and sex ratio. Both graphs show multiple points for each individual, accounted for using mixed effects models with individual identity as a random factor. The relationship between bound cortisol and sex ratio was significant (*P* = 0.030), while glucose was not correlated with sex ratio in this study (*P* = 0.836).

Overall, we observed a correlation between bound cortisol and sex ratio (χ^2^_1_ = 4.00, *P* = 0.046), with the relationship depending on timing of the sample during the breeding period (sex ratio*sample: χ^2^_1_ = 10.48, *P* = 0.015). Bound cortisol was significantly higher for male-biased litters during gestation (β = 106.81±43.28 ng/mL, *df* = 12, *t* = 2.46, *P* = 0.030; Intercept: 47.82± 37.41 ng/mL, *df* = 13, *t* = 1.28, *P* = 0.224; Fig. 2A), but not lactation (χ^2^_1_ = 0.003, *P* = 0.954). This relationship remained significant after removing several points identified as potential outliers using two-sided residual tests and diagnostics plots (α = 0.05), and during early gestation using a non-parametric test (Spearman’s ρ = 0.679, *P* = 0.011). There were similar, non-significant, increases in sex ratio with total plasma cortisol (χ^2^_1_ = 3.21, *P* = 0.073), but no correlation between FGMs and sex ratio (χ^2^_1_ = 0.03, *P* = 0.867).

We observed no relationship between glucose and sex ratio (χ^2^_3_<0.001, *P* = 0.992), including during gestation alone (χ^2^_3_ = 0.04, *P* = 0.836; Fig. 2B). Similarly, no relationship between testosterone and sex ratio was observed during the breeding season overall (χ^2^_1_ = 0.21, *P* = 0.648), though for early gestation alone, the reduced number of females for which we had testosterone values who also reared litters (n = 6) prevented any statistically meaningful comparisons.

## Discussion

In this study, we examined competing physiological mechanisms for ASA in a free-living rodent, *U. richardsonii*. Our data corroborate previous work in this population [25], finding both a positive relationship between cortisol and sex ratio during gestation, and a negative relationship between sex ratio and litter size in our focal females. While correlational, the present data do not support a glucose-based mechanism for ASA in this species, but rather suggest a potentially complex relationship between cortisol, testosterone, and embryonic mortality.

Total cortisol, bound cortisol, and free cortisol, were all highly correlated (*P*<0.001). However, free cortisol did not change through breeding, while bound and total cortisol, and the relationship between them, varied considerably (Table 1). Relatively stable free cortisol was also reported for female Arctic ground squirrels (*Urocitellus parryii*) in different breeding states [47], and although considered the biologically active fraction of cortisol in circulation ([27], but see [26], [48]), was unrelated to any of the reproductive metrics investigated in this study. In contrast, several relationships with both total and bound cortisol and variables of interest were uncovered.

First, higher total cortisol during gestation was associated with smaller litters, consistent with previous work suggesting a GC-mediated mechanism inducing embryonic mortality [18], [25], [49]. Total cortisol was also positively correlated with testosterone during gestation, itself negatively associated with litter size. While it is difficult to infer causality from these associations, the positive relationship between total and free cortisol and testosterone during gestation *and* lactation suggests a robust relationship between GCs and testosterone, independent of litter size. This relationship is consistent with a stimulatory effect of ACTH on the adrenal glands, the primary source of testosterone in female mammals [36], [50].

Whether total cortisol or testosterone during gestation are responsible for reductions in litter size – or vice versa – remains unclear; smaller litter sizes contained a higher proportion of male offspring, themselves a potential source of elevated testosterone in pregnant females (e.g. [51], [52], but see [53], [34]). However, contrary to the latter, there was no indication of a relationship between maternal testosterone and sex ratio during gestation in our study. This finding should be interpreted cautiously, since we could not restrict our analysis of these traits to early gestation, nearer the time when previous studies have reported a relationship, due to diminished sample sizes [23], [29]. Nevertheless, elevated testosterone has been shown to reduce fetal growth [54] and placental amino acid transport (but not glucose transport, [55]) providing an avenue through which T could affect litter size, as observed here.

We did find evidence that higher bound cortisol during gestation was accompanied by male-biased sex ratios. This is consistent with previous findings in this population using gestational fecal corticosteroid metabolites (FGMs) – a relationship that we did not observe in this study. While our previous work used means from 2–19 fecal samples taken throughout gestation, here we collected fecal samples during blood sampling only (2 samples max. during gestation). In theory, numerous individual samples averaged over time could buffer variability in FGMs introduced by diet, activity levels, or other factors [56], thereby providing a more representative picture of individual female ‘stress reactivity’ during gestation. What is clear is that if cortisol in its bound form is operative in ASA in this species, it is not through circulating levels of maternal glucose during our measured time points; we did not detect a positive relationship between maternal glucose and sex ratio during gestation, or a negative relationship between glucose and litter size, as predicted by the glucose-metabolism hypothesis (see also [24]). This may in part reflect our inability to capture glucose or changes in glucose just prior to conception in this study, which may be a crucial component in the glucose-metabolism hypothesis [22], [23]. However, although we were not able to control for feeding or other factors that could affect circulating glucose in our free-living subjects, we did not observe a positive relationship between cortisol and circulating glucose, contrary to the underlying assumptions of the glucose-metabolism hypothesis [24].

Failing support for the glucose metabolism hypothesis during our measured time points, the nature of the relationships between cortisol, litter size, and sex ratio that we observed are unclear. Considerable evidence does support the hypothesis that developing embryos from a very early stage exhibit sex-differential strategies for growth and survival in the womb, and that these differences are, in part, mediated through GCs [57], [58]. The successful implantation of the trophoblast into the endometrial stroma requires the localized immunosuppressive activities of GCs [59], and placental development, uterine invasion, and vascular remodeling all appear to show marked differences between the sexes, including in their response to elevated cortisol [57]. In addition to the importance of the timing of GC exposure, many of the effects of GCs operate synergistically with other hormones, including progesterone (P), itself binding with high affinity to CBG [58]–[60]. While any mechanistic hypothesis derived from the present study is necessarily conjectural, temporally-specific, sex-differential responses to GCs – accompanied by synergistic and interactive effects of P and CBG – could explain how both bound cortisol and total cortisol might affect two reproductive traits in a polytocous species relatively independently.

The relationships between cortisol, testosterone, and litter size, as well as cortisol and sex ratio, support earlier work proposing that antagonistic interactions and sex-biased dispersal may be key factors driving ASA in this highly social species. Females without strong matrilineal kin clusters would be either pushed to the edge of the colony and at a greater risk of predation or, within the colony, would suffer greater intra-specific competition for space and resources. Both scenarios are commonly associated with increases in cortisol and testosterone, and would favor mothers who produce the dispersive sex (i.e. males). The objective of this study was to focus on several key physiological variables implicated in ASA, not its social and ecological drivers. However, insofar as the ‘Advantaged matriline’ hypothesis could offer a complementary, or even alternative explanation to the Trivers-Willard hypothesis in this system, further investigation in this direction is warranted.

Our findings highlight the importance of reproductive trade-offs (offspring number vs. sex), and the involvement of some (i.e. cortisol and testosterone) – but possibly not all (i.e. glucose) – physiological parameters with ASA in a free-living, polytocous species. While small sample sizes warrant a conservative interpretation of our data, the results from our repeated measurements support earlier suggestions that the timing of sampling can be a key factor in detecting a relationship between physiological measures and sex ratio [11], [23]–[25], [61]. Finally, the discrete effects we observed for bound and free fractions of cortisol suggest potentially novel avenues (e.g. progesterone, binding proteins) for further research into the physiological mechanisms underpinning the adaptive manipulation of offspring number and sex.
